# Assessment of pharmacokinetics for microvessel proliferation by DCE-MRI for early detection of physeal bone bridge formation in an animal model

**DOI:** 10.1007/s10334-017-0615-2

**Published:** 2017-03-30

**Authors:** Bernhard Neumayer, Eva Amerstorfer, Clemens Diwoky, Richard A. Lindtner, Elisabeth Wadl, Eva Scheurer, Annelie-Martina Weinberg, Rudolf Stollberger

**Affiliations:** 1 Ludwig Boltzmann Institute for Clinical Forensic Imaging, Universitätsplatz 4, 8010 Graz, Austria; 2 BioTechMed, University of Graz, Universitaetsplatz 3, 8010 Graz, Austria; 3 Department of Paediatric and Adolescent Surgery, Medical University of Graz, Auenbruggerplatz 34, 8036 Graz, Austria; 4 Institute of Molecular Biosciences, University of Graz, Humboldtstraße 50, 8010 Graz, Austria; 5 Department of Trauma Surgery, Medical University of Innsbruck, Anichstrasse 35, 6020 Innsbruck, Austria; 6 Department of Pathology, Clinical Center Klagenfurt, Feschnigstraße 11, 9020 Klagenfurt, Austria; 7 Institute of Forensic Medicine, University of Basel, Pestalozzistraße 22, 4056 Basel, Switzerland; 8 Department of Orthopedics and Orthopedic Surgery, Medical University of Graz, Auenbruggerplatz 5, 8036 Graz, Austria; 9 Institute of Medical Engineering, Graz University of Technology, Stremayrgasse 16/III, 8010 Graz, Austria

**Keywords:** Contrast agents, Animal model, Physis

## Abstract

**Objectives:**

Bone bridge formation occurs after physeal lesions and can lead to growth arrest if not reversed. Previous investigations on the underlying mechanisms of this formation used histological methods. Therefore, this study aimed to apply a minimally invasive method using dynamic contrast-enhanced MRI (DCE-MRI).

**Materials and methods:**

Changes in functional parameters related to the microvessel system were assessed in a longitudinal study of a cohort of an animal model applying a reference region model. The development of morphology of the injured physis was investigated with 3D high-resolution MRI. To acquire complementary information for MRI-related findings qRT-PCR and immunohistochemical data were acquired for a second cohort of the animal model.

**Results:**

The evaluation of the pharmacokinetic parameters showed a first rise of the transfer coefficient 7 days post-lesion and a maximum 42 days after operation. The analysis of the complementary data showed a connection of the first rise to microvessel proliferation while the maximum value was linked to bone remodeling.

**Conclusion:**

The pharmacokinetic analysis of DCE-MRI provides information on a proliferation of microvessels during the healing process as a sign for bone bridge formation. Thereby, DCE-MRI could identify details, which up to now required analyses of highly invasive methods.

## Introduction

Premature physeal bone bridge formation can be induced by a variety of physeal insults including trauma, infection, radiation, ischemia, thermal injury, Blount disease, steroid use, as well as, iatrogenic and unknown etiologies [1]. Trauma in general including iatrogenic trauma from metal implantation is considered as the leading cause for formation of a physeal bone bridge [2, 3]. Bone bridges may be reversible when they are small, but can also trigger full or partial premature physeal closure due to incomplete decomposition and therefore may lead to full or partial growth arrest causing bone length discrepancy, axis deviation or joint deformity [4–7]. Clinically, they are detected by X-ray examinations, which are either routinely conducted to document fracture healing or initiated in patients with posttraumatic bone length alterations or deformities. MRI is then used to document the full size of the bridge and help in defining further management [1, 4, 8, 9].

A severe physeal bone bridge alters normal longitudinal bone growth and requires either surgical resection and filling up the lesion with autologous fat tissue as remedial measure or it is managed by contralateral epiphysiodesis or corrective osteotomy [1, 5, 6, 9]. Furthermore, stem cell therapy [10] and autogenous cultured physeal chondrocyte transplantation [11] have been recently investigated experimentally as novel therapeutic option. In contrast, minor bone bridges usually remain concealed and disappear during further growth, without impairing normal longitudinal growth [6, 7, 12]. Physeal bone bridge formation has been documented histologically [13–18] and by MRI [15, 16, 18] in various experimental animal models. Vascularization of the physeal lesion has been reported to precede physeal bone bridge formation [15, 19]. Along with invading vessels from surrounding epiphyseal and metaphyseal bone, mesenchymal osteoprogenitor cells emerge into the physeal lesion site and are attributed to deposit bone which eventually leads to bone bridge formation [15, 19]. The hypothesis that increased blood supply at the injury site is connected to the creation of physeal bone bridges as blood vessels and vascular invasion are fundamental for the development of bone tissue was further recently supported by the detection of increased angiogenic factor expression within the physeal injury site prior to bone bridge development [13, 20]. However, the underlying pathomechanism inducing angiogenesis followed by formation of a physeal bone bridge remains unexplored. The detection of an increase in blood supply in a physeal lesion could therefore allow an early identification of bone bridge formation and while the determination of angiogenic expression factors requires tissue samples, magnetic resonance imaging is potentially capable of providing this information non-invasively.

Dynamic contrast enhanced MRI (DCE-MRI) is sensitive to functional parameters of the microvessel system. It is therefore a tool to characterize angiogenesis and microvasculature and is widely used for the analysis of tumors [21–23]. The identification of pharmacokinetic parameters should therefore allow following the time course of microvessel-related functional changes during tissue remodeling on an injured physis.

Typical models for the identification of pharmacokinetic parameters  [24] require information on the time course of tissue contrast agent (CA) concentration and on the CA concentration within a feeding artery—the arterial input function (AIF). The simultaneous acquisition of both characteristics, i.e. the coverage of the AIF and, in this case, the physeal lesion in the same field of view (FOV), is very challenging and determines the limits for signal-to-noise ratio (SNR), contrast behavior and temporal or spatial resolution  [25–27].

For investigations where no adequate arterial input function is available in the field of view population-based averaged AIFs [28–30] or model-based arterial input functions  [31] can be applied to avoid a decrease in SNR or temporal resolution by modifying the FOV. A drawback of these methods is, however, that inter-individual variations between subjects or variations of the AIF due to manual CA administration cannot be covered. A different approach is to determine the CA dynamics of a reference tissue with known pharmacokinetic behaviour within the field of view. These dynamic data can then be used to apply a reference region model (RRM) which allows for the quantification of the pharmacokinetic parameters of a tissue of interest without the direct measurement of or assumptions on the AIF [32, 33].

The aim of this study was to investigate pharmacokinetic parameters of the microvessel system of a physeal lesion in correlation to bone bridge formation. These parameters are typically increased in tissues with enhanced metabolism and related to permeability surface product, blood flow and also blood volume. The investigation of these parameters should support the hypothesis that changes of the microvessel system are basic mechanisms associated with bone bridge formation and can provide quantitative measures for an early identification of this development.

The investigation of physeal lesions in a rat model using DCE-MRI required a very high spatial resolution and the application of a reference region model. As a complementary independent measure the results of the MRI analyses were compared to histologic findings and expression rates of genes related to vascular growth and oxygenation provided by quantitative real-time reverse transcription polymerase chain reaction data (qRT-PCR), which were acquired for a second cohort of animals.

## Materials and methods

### Animals

All animal experiments were conducted under animal ethical respect and were authorized by the Austrian Ministry of Science and Research. Seventy-seven male Sprague–Dawley rats with a weight of approximately 100 g and an age of one month were subjected to unilateral physeal lesion in general anesthesia using a standardized drill procedure [13, 14, 17, 34]. A longitudinal transepiphyseal lesion of 1.2 mm diameter was drilled through the proximal tibial physis, advanced from proximal by a median incision of the patella tendon.

For qRT-PCR analyses six animals were euthanized on days 0, 1, 3, 7, 14, 28 and 82 post-operation for sample dissection. For histological investigations five animals were sacrificed on days 1, 3, 7, 14, 28, 42 and 82; for the five animals designated for euthanasia on day 82 MR measurements were performed under anesthesia on the same seven time-points.

The investigation time-points were set in short intervals during the first month post-injury in order to closely follow bone bridge formation and changes in the microvessel system. The long-term time-points of 42 and 82 days were determined to follow further growth, physeal remodeling and to detect a potential disappearance of the bone bridge.

### MR measurements

**Fig. 1:**
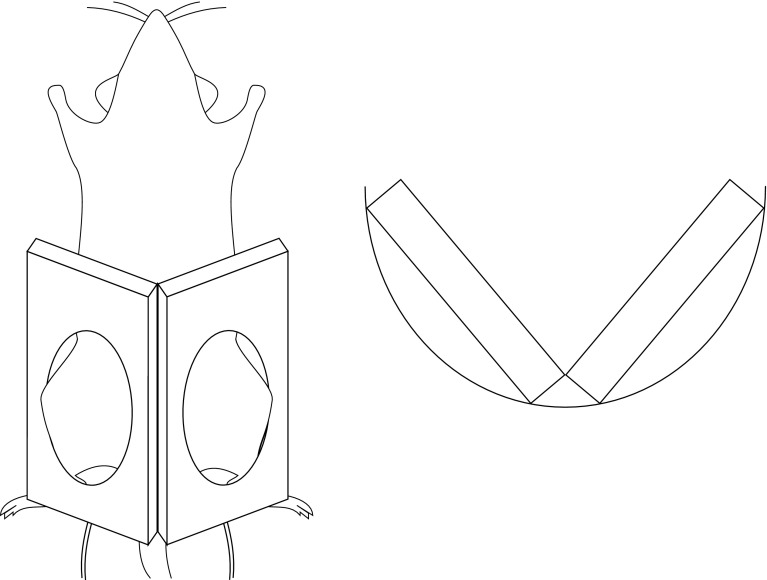
Arrangement of the surface coils for all MR measurements. *Left*: bottom view, *right*: arrangement of coils in the animal bed

Magnetic resonance imaging of the morphology and DCE-MRI were performed on a clinical 3 T scanner with 38 mT/m gradient strength (Tim Trio, Siemens AG, Erlangen, Germany). For enhanced image quality, especially suited surface received coils with a diameter of 18 mm (Rapid Biomedical, Rimpar, Germany) were used. Using an arrangement as shown in Fig. 1 a minimum distance to the rat knees and thereby a maximized SNR could be maintained for the complete duration of the study. Animals were placed in prone position with their knees in the center of the coils. This arrangement was positioned in a semicircular animal bed (Rapid Biomedical) providing a stable and reproducible assembly. As mentioned above, the measurements were performed on days 1, 3, 7, 14, 28, 42 and 82.

Morphological changes of the investigated area were imaged with a high-resolution 3D FLASH sequence (*T*_E_ = 7 ms, *T*_R_ = 100 ms, FA = 15, scanning matrix 256 × 256, FOV = 50 mm, THK = 0.72 mm) and coronal slice orientation related to the scanner’s reference system. Image quality was documented calculating ${\text{SNR}} = \mu_{\text{signal}} /\sigma_{\text{corr}}$, in which $\mu_{\text{signal}}$ is the average image intensity in a region of interest and $\sigma_{\text{corr}}$ is the standard deviation of a background region corrected for Rayleigh distribution. The corrected standard deviation is calculated $\sigma_{\text{corr}} = \sigma_{\text{Rayleigh}} /0.655$ with $\sigma_{\text{Rayleigh}}$ being the standard deviation measured in the image background region. Regions of interest for SNR determination were skeletal muscle and the physis.

The scans for DCE-MRI analysis comprised a single PD-weighted 3D FLASH reference sequence (*T*_E_ = 2.95 ms, *T*_*R*_ = 100 ms, FA = 5) scan and a *T*_1_-weighted dynamic 3D FLASH sequence (*T*_E_ = 2.95 ms, *T*_R_ = 8.09 ms, FA = 30) with a temporal resolution of Δ*t* = 13.32 s. This combination follows the basic idea of the approach proposed by Hittmair et al. for 2D FLASH acquisitions [35]. Postoperatively a double-dose injection (0.2 mmol/kg) of Gadovist (Schering AG, Berlin, Germany) was manually administered intravenously in the tail vein for DCE-MRI. The administration started after the acquisition of three baseline images for the calculation of pre-contrast longitudinal relaxation time *T*_1_; the entire protocol comprised 40 acquisitions. All scans for DCE-MRI were acquired in coronal orientation and featured a 205 × 256 matrix, a field of view of 50 mm and a slice thickness of 0.72 mm.

### Reference region model

Due to the absence of an adequate vessel providing an arterial input function in the FOV, blood supply related properties of the lesioned tissue were investigated by applying a reference region model  [33] to the acquired DCE data. Maps of the longitudinal relaxation time *T*_1_ were generated as proposed by Merwa et al. [36] according to Eq. (1)

1 $$ T_{1} (t) = - \frac{{T_{{R,{\text{DCE}}}} }}{{\ln \left( {\frac{{S_{\text{REF}} \sin (\theta_{\text{DCE}} ) - S_{\text{DCE}} (t)\sin (\theta_{\text{REF}} )}}{{S_{\text{REF}} \sin (\theta_{\text{DCE}} ) - S_{\text{DCE}} (t)\sin (\theta_{\text{REF}} )\cos (\theta_{\text{DCE}} )}}} \right)}}, $$

where *S* is the signal intensity in the image, $\theta$ is the flip angle and the subscripts REF and DCE denote reference scan and dynamic scan, respectively. According to Yankeelov et al. [33] and using the notation suggested by Tofts et al. [24], the relaxation rate $R_{1} \equiv 1/T_{1}$ of a tissue of interest (TOI) is related to the reference region by

2 $$ \begin{aligned} R_{{1,{\text{TOI}}}} (T) =\, & R \cdot (R_{{1,{\text{RR}}(t)}} - R_{{10,{\text{RR}}}} ) + R \cdot [K^{\text{trans,RR}} /v_{{{\text{e}},{\text{RR}}}} - K^{\text{trans,TOI}} /v_{\text{e,TOI}} ] \\ & \times \mathop \smallint \limits_{0}^{T} (R_{{ 1 , {\text{RR}}}} - R_{{ 1 0 , {\text{RR}}}} ){\text{e}}^{{( - K^{\text{trans,TOI}} /v_{\text{e,TOI}} )(T - t)}} {\text{d}}t + R_{{ 1 0 , {\text{TOI}}}} . \\ \end{aligned} $$

In Eq. (2) $K^{\text{trans}}$ is the transfer coefficient, $v_{\text{e}}$ is the extravascular extracellular volume, $R$ equals $K^{\text{trans,TOI}} /K^{\text{trans,RR}}$, $R_{10}$ is the native relaxation rate in absence of a contrast agent and TOI and RR denote the tissue of interest and the reference region, respectively.

The reference region for all investigations was skeletal muscle close to the area of the physeal lesion, i.e. the tissue of interest, and the pharmacokinetic parameters of skeletal muscle were set to $K^{\text{trans,RR}}$ = 0.045 min^−1^ and $v_{{{\text{e}},{\text{RR}}}}$ = 0.08  [37–39]. For all measurements, the subjects’ knees were located as close as possible to the center of the 3D slab to avoid different biases for the estimates of $R_{1}$ for RR and TOI due to $B_{1}$ inhomogeneities. The least mean squares routine for the parameter identification of $K^{\text{trans,TOI}}$ and $v_{{{\text{e}},{\text{TOI}}}}$ was realized in Matlab (TheMathWorks Inc., Natick, MA, USA).

### Histology

To obtain information on vascularisation complementary to the results of the analysis of the pharmacokinetic parameters, immunohistochemical analysis was performed for samples of the proximal epiphysis, physis and metaphysis of the tibial bone on the same days for a second cohort of animals.

Histological samples were fixed in methanol (100%) for 24 h and subsequently subjected to decalcification using Ethylenediamine Tetraacetic Acid (pH 7.0) for two weeks. Following a further fixation step in methanol (100%) for 12 h the samples were washed in PBS-sucrose 5% solution, covered with tissue freezing medium (Tissue Tek O.C.T. Compound, Sakura Finetek Europe B.V., The Netherlands) and cut into slices of 7 mm thickness.

To visualize angiogenesis of the physeal defect a collagen IV staining was used, as collagen IV represents a protein of the basal membrane of vessels, staining vasculature in a red/brown colour.

### Quantitative real-time reverse transcription polymerase chain reaction

Additionally to MR measurements and histological analysis, factors related to vascular growth and oxygenation were determined using quantitative real-time reverse transcription polymerase chain reaction analyses. The qRT-PCR analyses were performed for vascular endothelial growth factor a (VEGFa), which is a key parameter of angiogenesis [40] and hypoxia-inducible factor 1a (Hif1a) as a reporter for hypoxia.

For the analysis, physeal samples were dissected from the epiphysis and metaphysis under the microscope before storage in liquid nitrogen. The samples were homogenized using a tissue homogenizer and RNA was extracted using TRIZOL (Gibco BRL). Total RNA samples (2.5 mg) were reverse transcribed at 42 °C for 15 min using random hexamer priming. To establish quantitative expression profiles for VEGFa and Hif1a, commercially available, pre-optimized real time-PCR assays (Assay On Demand, Applied Biosystems) were used. Three reference genes (internal controls) were used for relative quantification. Normfinder [41] software was used to identify the optimal reference gene for internal normalization from a set of house-keeping genes. Triplicates were averaged and data was normalized to the house keeping gene *β*-actin of day 0.

### Statistical analysis

Statistical analysis was performed in SPSS 22 (SPSS Chicago, IL). Differences between results were tested for significance applying a post hoc Bonferroni test and a *p* value threshold of 0.05 for statistical significance; a p-value below 0.01 was considered highly significant.

## Results

**Fig. 2:**
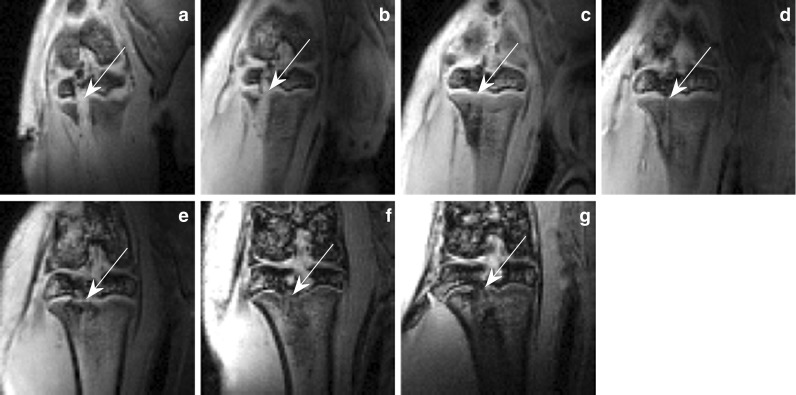
High resolution, non-contrast-enhanced MR Images of the lesioned area (*arrow*) in the tibial physis of one subject on days **a** 1, **b** 3, **c** 7, **d** 14, **e** 28, **f** 42 and **g** 82 post-lesion (images were brightened for a better view of the anatomy). The formerly disrupted area looks closed on day 7 but on day 28 it is clearly disrupted again

Figure 2 shows the high resolution and the excellent contrast of the morphological images acquired with the setting described above. The scans show the changes of the lesioned area of the physis (marked by arrows) of one representative animal for all 7 time points of the study and document the formation of bone bridges: On days 1 and 3 after the drill procedure, the drill mark causing physeal interruption is clearly visible. On day 7 the physeal interruption caused by the drill mark disappears leaving a homogenous high signal physis. A first low-signal bone bridge can be seen as early as on day 14 and progressively becomes more defined as a larger low-signal interruption within a high signal physis. These interruptions did not dissolve until the end of the study. SNR values showed an observable decrease after two weeks: skeletal muscle exhibited an average SNR of 82 in the first 14 days and 76 afterwards. For the physis, the SNR dropped from 72 to 36 on average.

**Fig. 3:**
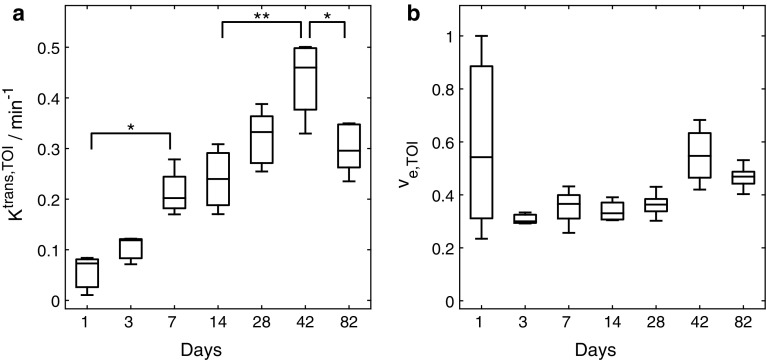
Results for **a** *K* ^trans^ and **b** *v*_*e*_ (*n* = 5 for each day). *Horizontal bars* indicate the median, the 25th and 75th percentiles. The values for the transfer coefficient start to increase after 3 days and reach their maximum on day 42 post-operation. The volume of the extravascular extracellular space is increased on days 1 and 42 but does not show statistically significant changes. Statistical changes are marked with *p < 0.05 and **p < 0.01, respectively

The analysis of the reference regions of all days and subjects yielded a relaxation rate of 0.82 ± 0.17 s^−1^. The results for the transfer coefficient and the extravascular extracellular volume provided by the reference region model are shown in Fig. 3: on day 1 post-operation $K^{\text{trans}}$ is at a minimum and $v_{\text{e}}$ is notably high; however, with strong variations. On day 3 a non-significant increase of the transfer coefficient accompanied by a decrease in $v_{\text{e}}$ is evident. The value of $K^{\text{trans}}$ on day 7 is significantly increased compared to day 1 and reaches its maximum on day 42 post-operation (highly significant different from day 14) with a concomitant increase of the extravascular extracellular space. Between the results of days 7, 14 and 28 no statistically significant differences exist. The value for the transfer coefficient decreased significantly from day 42 to day 82 accompanied by a decrease of $v_{\text{e}}$. For the entire study the results for $v_{\text{e}}$ did not show significant differences between measurement days.

**Fig. 4:**
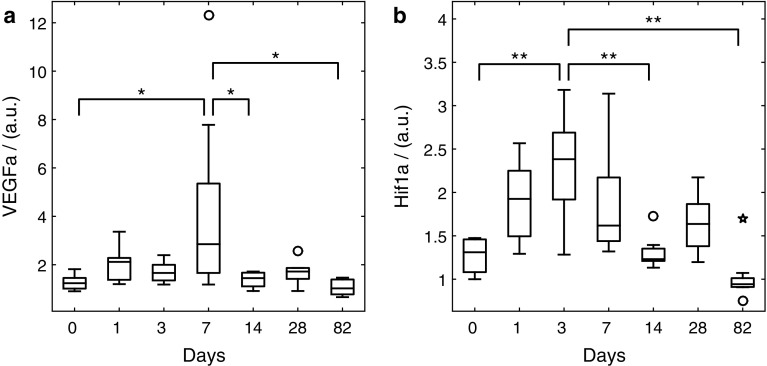
Quantitative real time reverse transcription PCR results for **a** VEGFa and **b** Hif1a (*n* = 6 for each day). *Horizontal bars* indicate the median, the 25th and 75th percentiles, minor outliers are displayed with a *circle*; major outliers with a *star*. Maximum vascular growth occurs on day 7 (VEGFa), forgone by maximum hypoxia on day 3 (Hif1a). Statistical changes are marked with *p < 0.05 and **p < 0.01, respectively

Concerning factors of vascular growth, a first visible but non-significant increase occurs on day 1 for VEGFa as an immediate reaction to the operation (Fig. 4). The only significant difference for VEGFa can be observed on day 7 post-lesion which shows increased values compared to the baseline (day 0) and days 14 and 82. The expression of Hif1a increases from day 1 on with maximum values on day 3 yielding a highly significant increase compared to days 0, 14 and 82.

**Fig. 5:**
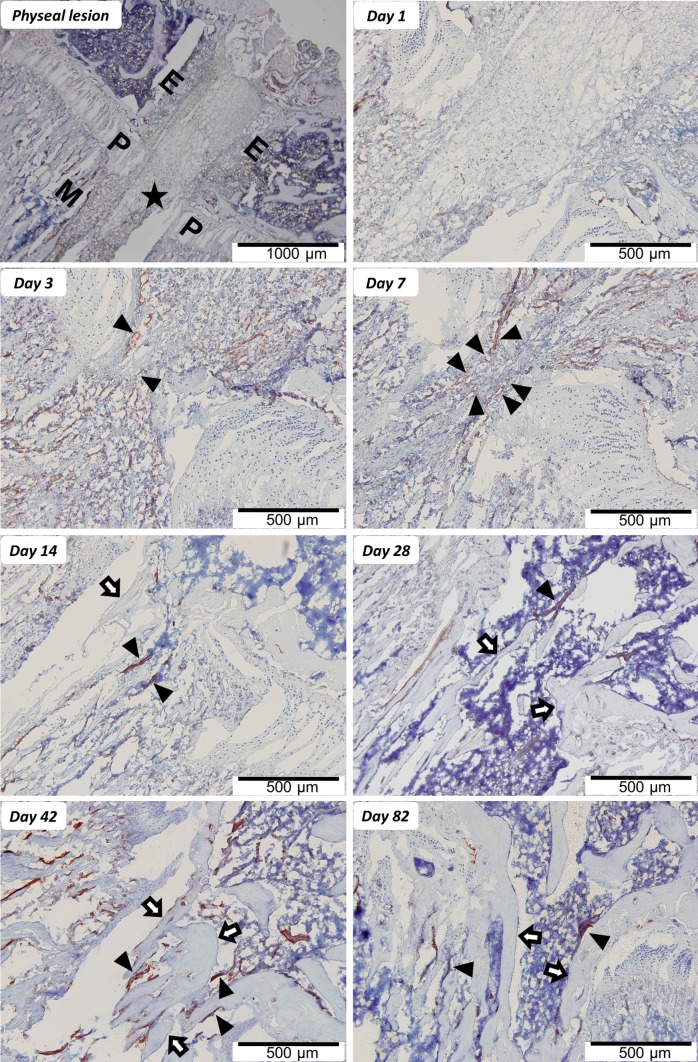
Immunohistochemical expression pattern of collagen IV demonstrating vascular ingrowth preceding bone bridge formation at the physeal lesion site. “Physeal lesion” shows an overview of the physeal lesion (*filled star* lesion, *P* physis, *E* epiphysis, *M* metaphysis of the tibial head). In the remaining images collagen IV-positive vascularization, stained *red/brown*, is highlighted by *arrowheads* (*filled pointer*), while *arrows* (*white arrow*) mark bone fragments which eventually form a bone bridge. The physeal lesion is postoperatively filled by hematoma without obvious vascular demarcation (day 1). Day 3 shows first capillaries ingrowing from surrounding hematoma into the physeal lesion site (indicated by *arrowheads*). The maximum number of capillaries pervading the physeal lesion are seen on day 7. Day 14 shows bony trabeculae traversing the physeal defect, maturing to a bone bridge connecting the epiphysis with the metaphysis by day 28 (marked by *arrows*). By day 42 the bone bridge consists of lamellar bone and is accompanied by distinct vessels, which decline with further maturation of the bridge (day 82)

Finally, histological investigations additionally showed the development of microvasculature in the physeal defect. Figure 5 shows collagen IV-stained histologic slices of days 1, 3, 7, 14, 28, 42 and 82 of the physeal lesion. On day 1 the staining showed no vascularisation. On days 3 and 7 post-operation the staining clearly displays the presence of capillaries (marked by arrowheads). After two weeks, first bony trabeculae (collagen IV-negative and marked by arrows) are observed within the physeal defect site. At this time-point collagen IV-positive vessels appear to be more structured compared to days 3 and 7. On day 42 the defective area is filled with lamellar bone fragments, which are surrounded by vessels. On day 82 post-operation the number of collagen IV-positive vessels appear reduced compared to day 42.

## Discussion

In order to detect bone bridge formation at an early stage using a minimally invasive method a reference region model was applied to DCE-MRI data of injured physes to analyze the changes of the microvessel system of this tissue. Furthermore, the changes of the pharmacokinetic parameter $K^{\text{trans}}$ over time were compared to qRT-PCR data and results of immunohistochemical analyses and the development of bone bridges was observed using high-resolution MRI.

The performed MRI studies show significant and clear time dependent changes of the assessed model parameter $K^{\text{trans}}$. This supports the hypothesis that changes of the blood supply are a potential mechanism associated with bone bridge formation of injured physes and permits a detection of the formation even before it becomes visible in MR imaging data.

Although this study was performed on a whole-body system, high quality morphological images could be obtained by using a dedicated receiver coil and by optimizing the parameters of the 3D gradient echo sequence. The used setting provided images with a voxel size of 0.19 mm × 0.19 mm × 0.72 mm, which allowed for an identification of the relevant image details. For the second half of the study the image quality was noticeably decreased. This is owed to the growth of the animals causing an increase in the distance between the surface coils and the region of interest, which consequently lead to a decrease of the SNR of the acquired data. However, the sharp decrease for the SNR of the physis after 2 weeks is also caused by a diminished signal intensity of the region of interest due to the animals’ age. This is also reflected by the much less pronounced decrease in SNR for skeletal muscle. The images did not suffer from susceptibility artifacts; however, for higher field strengths this may be the case, as reported by Taha et al.  [42], who found a RARE approach to be superior to FLASH at 9.4 T.

The high quality of the morphological scans provided a good insight into physeal bone bridge formations throughout the whole study. As was to be expected, the lesioned area can be clearly identified shortly after the application of the drilling procedure and the refillment of the injury was largely completed on day 7 post-operation. By only investigating image data for this day, this refillment could be accredited to healing processes closing the physeal lesion. While a remaining damage of the tibia was still visible, the formerly lesioned area of the physis was homogeneously closed and the isointensity of the filled lesion compared to the surrounding physis suggests a refillment with cartilaginous tissue.

First changes in the investigated area become slightly visible in the MR images on day 14 but without additional information these changes cannot be positively assigned to a bone bridge formation. However, the discontinuity of the physeal area on day 28 is a strong indication for bony activity. This new interruption of the physis occurred in all examined subjects indicating a strong connection between injuries across the physis and bone bridge-induced defective healing, as suggested in the literature  [13, 15, 17, 34]. The onset of bone bridge formation is in good accordance with findings of a recent study investigating the efficacy of MRI for the detection of changes in bone morphology  [42].

The specific requirements of this animal study lead to a moderate temporal resolution of the dynamic scan. This and the lack of a feeding artery in the investigated area required the application of an RRM to acquire pharmacokinetic parameters. The reference region was skeletal muscle and the average relaxation rate of the reference regions of all days and subjects is in good agreement with literature values  [43, 44]. The analysis of the pharmacokinetic parameter $K^{\text{trans}}$ revealed changes in the investigated area, which were not visible in imaging data. These changes can be attributed to the underlying mechanisms of the formation of bone bridges, which did not become visible in the morphological images before day 14 and could not be clearly identified before day 28 post-operation. On days 1 and 3 post operation the lesion is mostly filled with blood, thus $K^{\text{trans}}$ cannot be reasonably determined. Especially on the first measurement day the development of blood vessels is expected to be still very low, which hinders a meaningful parameter identification. This, for instance, is reflected in the quite strong variance of the values for $v_{\text{e}}$. However, from day 3 onwards the fitting routines provide robust, i.e. less scattering, results and the increase of $K^{\text{trans}}$ suggests a proliferation of microvessels.

While it is not completely clear whether such an establishment of a microvessel structure triggers a defective healing and thereby hinders a *restitutio ad integrum*, it can be considered as a first sign for the formation of bone bridges. This is a remarkable finding, since prior to this study date neither histologic analysis nor MR image data could unambiguously detect newly formed bone tissue. Histology could confirm the presence of bone fragments in the lesioned area  [13] but it is still disputed if these bone fragments within a fracture site lead to the formation of bone bridges, as according to Xian et al.  [17], an irrigation of the drill track had no measurable effect on the development of osseous matter. Therefore, the determination of $K^{\text{trans}}$ allows for an earlier detection of bone bridge formation.

After day 3 post-operation the transfer coefficient indicates a steady increase. The maximum value of $K^{\text{trans}}$ on day 42 reflects the blood supply of bone bridges, which could be clearly detected in the morphological images at that time.

The volume of the extravascular extracellular space did not provide additional information. The decrease at the beginning of the study could be interpreted as a sign for increased blood supply; however, it was reported that the RR estimate of $v_{\text{e}}$ and the estimate produced by the standard method with known AIF are not correlated  [45]. Therefore, and since the changes of this parameter were only statistically non-significant the course of this quantity was not investigated further.

The analysis of collagen IV-stained histology data confirmed the presence of vascularity from day 3 onwards, which coincided with an increase of $K^{\text{trans}}$. After two weeks the stained vessels appear less scattered but greater in size and first formations of bone bridges can be detected. This is followed by another increase of $K^{\text{trans}}$ until day 42 followed by a decrease between days 42 and 82, which coincides with a seemingly reduced staining. The voxel based integrative parameter *K* ^trans^ show a smoother development with less variation in comparison to parameter from histological samples.

The increased expression of VEGFa on day 7 is preceded by a rise in the expression of Hif1a with its maximum on day 3. VEGF expression has been reported to be induced by hypoxia [46]. Furthermore, the heterodimeric basic helix-loop-helix protein Hif1 has been attributed to directly activate VEGF transcription in hypoxic cells, implicating Hif1a to play a pivotal role within this cascade [47, 48]. Although, VEGF and Hif1a were not measured on protein levels in our study, we suspect that, based on our qRT-PCR results, the changes of the microvessel system and the increased values of $K^{\text{trans}}$ occurring on day 7 were most probably triggered by hypoxia within the physeal lesion site. The scattered VEGFa expression observed primarily on day 7 is presumably associated with the relative small sample size, where a short increase of expression towards the randomly selected day 7 may be hard to detect exactly. The statistical outlier of day 7, however, may be due to a contaminated sample by surrounding tissue.

Comparing the results of MR imaging and the pharmacokinetic analyses to the complementary data it can be stated that the increase in $K^{\text{trans}}$ corresponds to a filling of the physeal injury with vasculature. The data also shows that the non-significant increase of $K^{\text{trans}}$ between days 7 and 14 coincides with a return of VEGFa expression to its initial value on day 14. However, it is also notable that qRT-PCR results reveal a rather pronounced variance in the data while the pharmacokinetic analysis provides a clearly identifiable trend. The results suggest that the combination of high-resolution MRI with a pharmacokinetic analysis allows to identify bone bridge formation at an early stage, while being only minimally invasive.

Currently, Gadolinium-based contrast agents are not generally used for the detection of physeal bone bridges; however, contrast-enhanced measurements are already applied for different disorders, such as the Legg–Calvé–Perthes disease [49]. Therefore, we expect our method to be generally applicable to children, which would provide a quantitative measure for changes in the micro-vessel system associated with the formation of bone bridges at an early stage. The detection of changes in *K* ^trans^ was at least 7 days earlier than the detection of first morphological signs for bone bridges. This time period may translate from the specific animal model to a duration between 6 and 19 months for humans [50, 51], which can be particularly important in cases with concealed physeal bridging. Clinically, many physeal bone bridges may not become evident until months or years have passed and growth disturbances have developed [1]. This highlights the necessity for early recognition of physeal bone bridge formation, which subsequently aids surgical management in order to restore potential growth to the bone.

## Conclusion

In this study, we used DCE-MRI on a clinical system to assess parameters related to blood supply in an animal model in order to investigate the possibility to detect the formation of bone bridges using a minimally invasive method. We conclude that a transphyseal lesion is associated with increased values for $K^{\text{trans}}$ which could be observed from day 3 onwards using DCE-MRI. The increase of the transfer coefficient coincides with a presence of vascularity, which appears to trigger the consecutive bone bridge formation and thereby may hinder a *restitutio ad integrum* of the physeal defect.

MR measurements did not only document the formation of bone bridges but additionally provided quantitative information on tissue development and microvessel proliferation in the lesioned area. The analysis of the DCE data enabled an indirect detection prior to visibility in morphology images, which may help in preventing growth disturbances in children by allowing therapeutic intervention early in course. This finding, however, still needs verification in a human study.

## Abbreviations

*K*^trans^: Transfer coefficient
*v*_e_: Extravascular extracellular volume
AIF: Arterial input function
CA: Contrast agent
DCE: Dynamic contrast enhanced
FA: Flip angle
FLASH: Fast low angle shot
FOV: Field of view
Hif1a: Hypoxia-inducible factor 1a
PD: Proton density
RARE: Rapid acquisition with relaxation enhancement
RNA: Ribonucleic acid
RR(M): Reference region (model)
SNR: Signal-to-noise ratio
TOI: Tissue of interest
VEGFa: Vascular endothelial growth factor a
